# Medical Care or Disciplinary Discourses? Preventive Measures against the Black Death in Late Medieval Paris: A Brief Review

**Published:** 2017-03

**Authors:** Yong Jin HONG, Sam Hun PARK

**Affiliations:** 1. Institute for Human Urbanities, University of Seoul, Seoul, Korea; 2. Asia Contents Institute, Konkuk University, Seoul, Korea

**Keywords:** Black death, Medical treatment, Middle ages, Knowledge/power

## Abstract

**Background:**

This paper examined the political and social implications of the *Compendium de epidemia* prescription written by the Masters of the Faculty of Medicine of the University of Paris in the mid-14th century during the Black Death. This study aimed to examine how the effects of power as a discourse owned by medical knowledge are revealed.

**Methods:**

This paper outlines the composition of the contents based on the 1888 edition edited and translated by Émile H. Rébouis and notes the features of the prescription examined by the existing study of medical history rather than the causes of diseases.

**Results:**

*Compendium de epidemia* seems to have been written primarily for the royal family and nobles who ordered them when looking at prescription-related technologies. At the same time, under the influence of Islamic-Arabic academia, it clearly distinguishes the world of faith and the world of academia (intelligence), explaining the pathogenesis and infection pathways based on causality. The onset substrate is due to heat and humidity, and the prescription is to prevent the two from overdoing in the body. In particular, issues related to heat are criticized in connection with the value of life of *knight-noblesse*. This is in response to political criticism of the ineffectual French royal family and nobility at the beginning of the Hundred Years’ War and shows why this tract sets the *utilitas publica* at the forefront as an important purpose.

**Conclusion:**

The conclusion has shown how medical knowledge produced on the Black Death pandemic how they function as discourses that have a sort of power effect on the value of life of knight-noblesse. It is necessary to conduct if these phenomena can be found in other contemporary medical writings.

## Introduction

The Black Death, which lasted from 1347 to 1350, devastated the entire Europe in the late middle ages. The Black Death, which began in Sicily in Oct 1347, reached southern France in just two to three months, and in the late summer of 1348, it reached Paris, the capital of France, and swept the entire French region in the summer of 1349. As is already well known, the plague reduced the European population to less than half of its original size and caused crises and changes in socio-economic and cultural terms (1–4). Europeans were helpless about this new infectious disease. However, European medical scientists analyzed the cause of Black Death based on their own knowledge and proposed prevention and treatment prescription for it.

In France, there were six reports of the Black Death-related diagnoses and prescriptions written at that (Table 1).

**Table 1:** Six reports at the outbreak of the Black Death (5)

**N.**	**Author**	**Title**	**Date**
1	Pierre de Damouzy	*Tractatus de epydemia*	1348
2	Faculty of Medicine of the Univ. Paris	*Compendium de epidemia*	1348
3	Alphonso of Cordova	*Epistola et regimen Alphontii Cordubensis de pestilentia*	1348
4	Anonym of Montpellier	*Tractatus de epidemia*	1349
5	Anonym	*Causa epydimie et preservation ejusdem*	1348–49?
6	Anonym	*Compendium breve contra epydimiam*	1348–50?

At first, Pierre de Damouzy, a physician in the Reims region studied at the Paris School of Medicine, wrote a report on the Black Death in preparation for the impending spread of the disease to this area. The second report of the Black Death pandemic, which is the theme of this paper, was written in Oct of the same year by the Masters of the Faculty of Medicine of the University of Paris at the request of the French King, Philip VI (1328–1350). The third report was written by a medical scientist, Alphonso from Cordova, at Montpellier, France. The rest of the three reports briefly summarized the above-mentioned reports on pandemic analyses and prescription written by anonymous medical scientists in later years. If the first report was the earliest written one in France, then the second report showed greater importance in terms of academic authority.

*Compendium de epidemia*, the Black Death tract of the University of Paris, received great attention from the medical profession. The Latin manuscripts (especially BNF, lat. 11227) were translated into modern French together with interpretations of the content of the *Compendium de epidemia* (6).

Recently, more precise were conducted and complex analyses of the *Compendium* with respect to medical history while comparing with the medical knowledge of the time (7–10). Their work is very important in terms of the fact that it analyzes how each report is correlated to one another based on a systematic analysis of all the medical knowledge and discourses circulated in the medical field until the beginning of the 14th century.

*Compendium de epidemia* largely consists of a description of the causes of the illness and a prescription for it. Thus, analysis concentrated on the interaction between influential relationships and medical discourses, focusing mainly on explaining the causes of illness.

In contrast, this paper focuses on the second part, dealing with prescribing, especially changes in lifestyle and medical care to prevent Black Death. Moreover, it intends to examine what its political and social implications are.

## Methods

### Source

The source used for this analysis is based on the modern editions and translations of E. H. Rébouis (6).

### Analysis of the text structure

The composition of the *Compendium de epidemia* is as follows (Table 2).

**Table 2:** Structure of the *Compendium*

**Part I**	Ch. 1 On the general and distant cause	
	Ch. 2 On the particular and near cause	
	Ch. 3 On the prognosis and symptoms	
**Part II**	Treatise 1 Preservative remedies By means of Diet	Ch. 1 On Choice of air and its Purification
		Ch. 2 On Exercise and Bath
		Ch. 3 On Food and Drinks
		Ch. 4 On Sleep and Wakefulness, Fast and Repletion, Accidents of the Soul
	Treatise 2 Medicinal remedies	Ch. 1 General remedies
		Ch. 2 Particular and proper remedies
		Ch. 3 Antidotes

As is well known, this report examines the cause of Black Death by dividing it into direct and indirect causes. Although it is not a proper diagnosis and prescription from the modern perspective, this book explains the Black Death using contemporary academic knowledge of the time, excluding most of the religious factors. Following the analysis of the cause, prescriptions are presented. What is interesting is that, unlike modern medicine, daily life health treatments such as dietary therapy are suggested first rather than medical remedies.

The matters intend to focus in this report are on the various preventive measures and medicinal products shown in Part II. Treatise 1, divided into four chapters, present precautions to prevent the Black Death in daily life, such as breathing, exercise, bathing, eating and sleeping. In the following Treatise 2, it suggests three kinds of medicines for preventing the Black Death. In particular, the ‘right life attitude’ proposed by the Paris University School of Medicine is not a simple description of knowledge, but rather has the nature of instruction and direction to follow. This raises the need to review the correlation between knowledge and power already pointed out by Michel Foucault, by applying them to this case (11).

## Results

### Causes of the Black Death

**Fig. 1:**
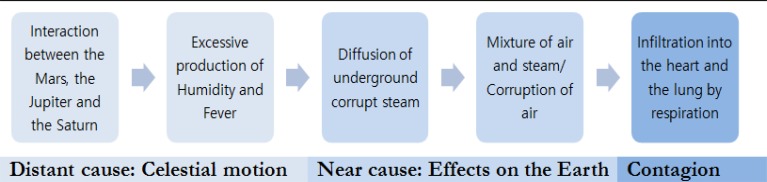
Causes and process of the Black Death

If saying that a distant cause is related to celestial phenomena, it indicates the near cause is related to the effect on the earth from such celestial phenomena. The analysis of causes is explained by a chain of causal relationships, regardless of religious terms (Fig. 1). In particular, the report points out that the Black Death is a plague caused by excessive fever and humidity. Therefore, people with these two temperaments are more likely to get sick.

### Preservative remedies

In Part II, the authors honestly acknowledge that there is no fundamental cure for the epidemic, and then offer a variety of possible ways to avoid the disease. As mentioned earlier, Part II is divided into Treatise 1 related to health therapy in daily life and Treatise 2 is related to medicines. First, the summary of Treatise 1 is as follows.

Chapter 1: Be careful of wetland air and winds blowing from the south, where the Black Death started; Purifying air by burning fragrant grass and dry firewood (ex. Juniper, Fraxinus, Rosemary, etc.); Air purification through fumigation (Aloe, Ambergris, Musk, etc.).
Chapter 2: If you are in the habit of exercising, then take a walk when the air is clear. Otherwise, exercise should be practiced indoors. Avoid a hot water bath that sags the body, but If not overweight (if having a substrate that is not hot and humid), the occasional bath will be very good.
Chapter 3: Avoid over-eating and excessive drinking. Avoid moisturized and heavy foods and eat light-weighed foods easily digestible.
  - Meat Recommended for Intake: Intake of meats from lambs, goats, rabbits, tender beef and various birds (chickens, partridges, pheasants, starlings). Add various spices and vinegar to the meat, rather than boiling water.
  - Meat Recommended Avoiding: Meats of cattle, pigs, deer, goat, all water birds, and fishes. These foods are difficult to digest, leaving the intake with a feeling of depressing and a heavy body.
  - Watery vegetables, fruits, soups and dairy products should be consumed in small quantities and the vegetables should be eaten with added spices and vinegar. The exceptions to this rule are sour fruits (Pomegranate, Lemon) and low moisturized fruits (Fig, Walnut).
  - Wine and water are to be ingested in a clear and clean state. For water, either drink spring water or in the state of being boiled or distilled. It is good to add vinegar.
Chapter 4: Get a good night’s sleep and avoid a nap during the day. Avoid over-eating, and relieve hunger with soybeans. Obese people should purify themselves with medicine. No excessive sex life. Avoid excessive anger, depression, anxiety, and maintain stability and an enjoyable life and practice a life of faith.

### Medicinal remedies

Chapter 1. Next, Treatise 2 suggests medicines that should be ingested to prevent the Black Death.
Chapter 1. Eliminate bad humor through bloodletting and purify the body. Drink syrup containing vinegar and honey.
Chapter 2. Preventive medicines are as follows.
  - Food as medicine: Vinegar, Sour milk, Garlic, Sorrel (Rumex acetosa), etc.
  - Only Medicine: *Armenian bole (bolus armenicus), Terra sigillata, Aagaricus, Tyriacum, Emerald, Aroma*-herbs etc.
Chapter 3. Antidotes: Preventive medicines, air purifiers, and pills made by mixing aloe and various herbs.

## Discussion

The Compendium is based on medical knowledge up to the beginning of the 14th century, against the Black Death epidemic of the time. However, it cannot be treated simply as a product of the medieval medical knowledge. Because the report itself is a social product and at the same time it reveals political and social realities of various contemporary issues besides medicine.

The first thing to note is that a very limited number of people read the report. First, this report was written in Latin indicates that those read and understand this text were limited to priests, including academics. Not only the elements of various medicines presented in the diversified remedies, such as the terms like court and obesity but also the names of various medicines, show that this report was written for the king and nobility, not for public. At the time, people who lived in mansions with courts and were likely to be obese were the wealthy classes, such as nobles, high priests, and *bourgeoisies*. In addition, the medicines offered in the *Compendium* are very expensive ones that are difficult for commoners to afford. In addition, here we can add the fact that Raymond Cazelles has already pointed out, through several sources and images, since Philip VI had an obese constitution (12). This report is written at the request of Philip VI of France, is prepared only for the King and the courtiers of Paris surrounding him.

Second, as mentioned earlier, this tract tries to explain the cause and prescription of the Black Death according to medieval academic logic. Of course, this logic does not extend beyond the limits of medieval times, and the will of God is the ultimate cause of the Black Death (P. I, Ch. 3, the last paragraph). In addition, devotional life of faith is mentioned in the lifestyle for prevention (P. II, T. 2, Ch. 4). Nevertheless, the *Compendium* does not directly associate religious faith with the Black Death, unlike the contemporary social phenomena such as the Jewish massacre or Flagellant of the same time. As much as the Black Death is God’s will, God also creates medicine, and the faith life is mentioned in connection with maintaining a cheerful mood to prevent illness.

The development of philosophy, science, and medicine in Europe from the 12th to the 13th centuries owed substantially to the Islamic-Arab world (13). This is the same in *Compendium,* and the Masters of the University of Paris is leaning heavily on the description frame of Ibn Sina (Avicenna) (14, 15). However, it is also important to note an attitude to distinguish between the faith and the reason. This brings us to the *Liber de causis* written by an unknown author (or Pseudo-Aristotle), which had a strong influence on Western European theology and philosophy (16). This book clearly separates the world into the sphere of the Eternity (*Aeternitas*) and the sphere of the Time (*Tempus*) for Creation and Changing, so-called theology and philosophy. Philosophy encompasses knowledge of man and nature, which establishes an independent system of knowledge separated from the field of theology and faith. This shows the contemporary philosophical tendencies of the first half of the 14th century that clearly distinguish between the divine and the human (secular). Furthermore, it corresponds to the political thought of the time, which seeks to identify Human Polity (*Respublica*) as Autonomous, distinct from the theological worldview.

On the other hand, the authors also reject the simple analogical relationship between the Macrocosm-Microcosm, commonly practiced at the time. There is different causal relationship acting between the motion of the celestial body and the change of the human body, which distinguishes astrology and medical science. Thus, in the *Compendium*, there is a clear distinction between the academia of the creator and the academia of the creatures, and a clear division or differentiation between the sub academia in the latter.

According to the causality of the world belonging to the time suggested by the *Compendium*, the strong heat and dry seasons formed in the celestial bodies affected the earth. These two properties raise the moisture that was deeply latent underground into the air and produced toxic air. The heat and humidity became the characteristics representing this toxicity. In addition, all the prescriptions and treatments that appear in Part II aim at avoiding or alleviating heat and humidity. The third point worth noting is that these medical diagnoses, prescriptions and precautionary measures are accompanied by critiques of a certain lifestyle and value of life for the aristocrats of the time.

The authors of the *Compendium* think that the air containing the heat and humidity comes from a near distance such as swamp or puddle and from a distance, originates from the southern provinces, early infected by the Black Death. Therefore, it suggests restraining the outdoor exercise and the hot water bath. One thing to note about the items related to a bath is that it is occasionally allowed for people who are not fat. Here, the authors link the constitution of fattening obesity with the combination of heat and moisture. This is more evident in chapter 3, which deals with food. Authors recommend to avoid damp foods in addition with overeating and overeating and to take light foods such as grains. In particular, here they divide the meat allowed to be consumed and the meat to be avoided, and this distinction follows the medieval classifications between heavy and light foods.

- Light meats: Mutton, Goat, Hare, Poultry, Birds, Beef (Tender part)
- Heavy meats: Beef (Old cow), Pork, Venison

There was a conflict between different health food dietary menus in the medieval ages (17). One was to encourage a “light” diet to make the body light, flexible and slimy, and the other was to encourage “heavy” meals for a strong and sturdy body. Moreover, this has to do something with the distinction of the medieval social classes between the priest and the secular. If the former, which the clergy usually recommend, was a diet that enhances the purity of the body, the latter, was the diet of strength and robustness that was favored by knights who had combat as a profession. The *Compendium*, of course, does not say anywhere that foods to boost vigorous power should be avoided. Moreover, the most emphasis is on the moisture among the elements of the food to be avoided. However, the vinegar, which appears most commonly as food material and medicinal ingredient in the entire article, shows that this article is concerned with heat as well as moisture.

If such analysis is reasonable, the *Compendium* contains a presupposition of confrontation between a light-weighted, pure diet and a heavy and energy-boosting diet (17). Moreover, since all those called academics or scholars in the middle ages were clergymen, the authors of *Compendium* used the Black Death as an opportunity to suggest that the priest’s diet was the proper diet that ought to be taken rather than a knight’s diet. These intentions of them are in line with the warning of obesity caused by so-called “heavy” foods. Furthermore, physical obesity leads to accidents of the soul, such as rage and sadness. To this end, the *Compendium* recommends maintaining pleasure and stability in various ways in life.

Is such medical knowledge simply aimed at treating and preventing diseases? In fact, it is not clear if there are other intentions of Masters, besides communicating medical knowledge. Nevertheless, when examining the contents of the *Compendium* in terms of socio-cultural implications and effects, it shows the criticism and timing strategy of clergy and intellectuals on the nobles (knights) who are monopolizing violence.

In fact, since the 10th century, priests have been trying to control and suppress violence of the knight-noblesse order in a variety of ways. In the 14th century, the discourses of medical knowledge and health maintenance began to imply the functions of violence control and restraint in the wake of the Black Death. The looks that the traditional knights had shown including heavy foods (or high-calorie foods), obesity (or sturdiness), and anger (or vigorous) were rated as bad for their health before they became moral problems. More specifically, the incident of the Black Death occurred in the context of the unprecedented State war and imposition so called “The Hundred Years’ War”. In other words, the *Compendium* is bound to the contemporary criticism of the temerity of the French aristocracy that had caused the devastating defeat, as well as the excessive taxation of the war (18–19). Moreover, at this level, this tract can be said to be oriented toward ‘public wealth (utility publica; bien public)’, as it reveals at the beginning of the book, even though it is aimed at kings and aristocrats (18–19). In fact, this term is significant that was spread all over the capital of France, Paris after the Assembly of estates, which was held in 1347 with the call for a reform of the kingdom (20).

## Conclusion

In the light of Michel Foucault’s argument that knowledge itself functions as a discursive power, the *Compendium*, which conveys medical diagnosis and prescription, also it contains the character of discourse power in the political and social contexts of the time. It is important to look at the socio-cultural implications of other Black Death tract published later at the time.

## Ethical considerations

Ethical issues (Including plagiarism, informed consent, misconduct, data fabrication and/or falsification, double publication and/or submission, redundancy, etc.) have been completely observed by the authors.
